# A Tumor-Feeding Artery Towards a Cardiac Glomus Tumor

**DOI:** 10.1016/j.jscai.2023.101263

**Published:** 2023-12-22

**Authors:** Deborah N. Kalkman, Frank van der Kley, Paul Dekkers, E. Wierda, Remko S. Kuipers, Hugo A. van de Klippe

**Affiliations:** a Department of Cardiology, Amsterdam UMC – location AMC, Amsterdam, the Netherlands; b Department of Cardiology, Leiden University Medical Center, Leiden, the Netherlands; c Department of Cardiology, Dijklander ziekenhuis – Hoorn & Purmerend, the Netherlands

**Keywords:** coronary steal phenomenon, glomus tumor, tumor-feeding artery

A 52-year-old male patient was referred to the outpatient cardiology clinic for atypical chest pain. No abnormalities were seen during physical examination. His medical history included hemorrhagic stroke due to catecholamine excess produced by glomus tumors, removal of 2 glomus tumors from his ear and neck, and video-assisted thoracic surgery for resection of a suspected mediastinal glomus tumor 2 years ago. Glomus tumors are mostly benign, well-vascularized mesenchymal neoplasms belonging to the group of paragangliomas. These tumors can produce hormones such as adrenaline, noradrenaline, and dopamine.^1^^,^^2^

**Figure 1:**
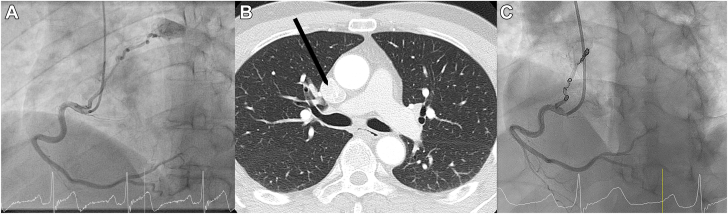
**Coronary angiogram and CT scan before coiling and coronary angiogram after coiling of the tumor-feeding artery.** (**A**) Coronary angiogram showing the right coronary artery with a fistula toward a structure near the roof of the left atrium. (**B**) CT scan showing the cardiac glomus tumor indicated with a black arrow. (**C**) Coronary angiogram showing the right coronary artery with the tumor-feeding artery after coiling.

Bicycle ergometry test showed no abnormalities. Transthoracic echocardiography showed a normal ejection fraction, and no wall movement or valve abnormalities. Cardiac computed tomography revealed a high coronary calcium score (600, >90th percentile for his age and sex) but was inconclusive for flow-limiting coronary stenoses. Therefore, he was referred for coronary angiography. Coronary angiography showed no significant obstructive coronary artery disease in the left main, circumflex artery, or the anterior descending artery. Injection of contrast into the right coronary artery showed a fistula, originating from the proximal right coronary artery toward the roof of the left atrium, visualizing a highly perfused round structure (Figure 1A).

After evaluation by the heart team, it was concluded that the tumor-feeding artery could be responsible for angina symptoms due to coronary steal syndrome. The highly vascularized structure was suspected to be a cardiac glomus tumor. In retrospect, the suspected cardiac glomus tumor could be seen on the computed tomography scan (Figure 1B). The heart team's decision was to perform percutaneous closure of the tumor-feeding artery with coiling. Three VortX Vascular Occlusion coils (Boston Scientific) were used (two 4.0 mm and one 5.5 mm). The patient underwent the procedure without complications. The result can be seen in Figure 1C.

In the outpatient clinic, the patient reported the disappearance of his angina symptoms, supporting the hypothesis of coronary steal syndrome. We hypothesize that the cutoff of blood to the tumor stopped the tumor from generating angina-like symptoms.

Coronary artery fistula has been found in the presence of, for example, myxomas of the heart,^3^ but only a few cases of cardiac glomus tumors have been described.^1^ To our knowledge, this is the first report of treatment of angina resulting from a tumor-feeding artery deriving from the right coronary artery toward a cardiac glomus tumor.
